# The Influence of Costimulation and Regulatory Cd4^+^ T Cells on Intestinal Iga Immune Responses

**DOI:** 10.1155/1998/75718

**Published:** 1998

**Authors:** Eva Gärdby, Dubrav Kagrdic, Martin Kjerrulf, Annakari Bromander, Michael Vajdy, Elisabeth Hörnquist, Nils Lycke

**Affiliations:** Department of Medical Microbiology and Immunology University of Göteborg Göteborg S-413 46 Sweden

## Abstract

It is thought that IgA B-cell differentiation is highly dependent on activated CD4^+^ T cells. In particular, cell-cell interactions in the Peyer's patches involving CD40 and/or CD80/CD86 have been implicated in germinal-center formation and IgA B-cell development. Also soluble factors, such as IL-4, IL-5, IL-6, and TGFβ may be critical for IgA B-cell differentiation *in vivo*. Here we report on some paradoxical findings with regard to IgA B-cell differentiation and specific mucosal immune responses that we have recently made using gene knockout mice. More specifically, we have investigated to what extent absence of CD4^+^ T cells, relevant cytokines, or T-cell-B-cell interactions would influence IgA B-cell differentiation *in vivo*. Using CD4– or IL- 4-gene knockout mice or mice made transgenic for CTLA4Ig, we found that, although specific responses were impaired, total IgA production and IgA B-cell differentiation appeared to proceed normally. However, a poor correlation was found between, on the one hand, GC formation and IgA differentiation and, on the other hand, the ability to respond to T-celldependent soluble protein antigens in these mice. Thus, despite the various deficiencies in CD4^+^ T-cell functions seemingly intact IgA B-cell development was observed.


*Developmental Immunology*, 1998, Vol. 6, pp. 53–60
Reprints available directly from the publisher
Photocopying permitted by license only

© 1998 OPA (Overseas Publishers Association)
N.V. Published by license under the
Harwood Academic Publishers imprint,
part of the Gordon and Breach Publishing Group
Printed in Malaysia

### The Influence of Costimulation and Regulatory CD4$^+$ T Cells on Intestinal IgA Immune Responses

EVA GÄRDBY*, DUBRAV KAGRDIC, MARTIN KJERRULF, ANNAKARI BROMANDER, MICHAEL VAJDY, ELISABETH HÖRNQUIST and NILS LYCKE[a]

*Department of Medical Microbiology and Immunology, University of Göteborg, S-413 46 Göteborg, Sweden*

(*Received 11 August 1996; In final form 15 June 1997*)

It is thought that IgA B-cell differentiation is highly dependent on activated CD4$^+$ T cells. In particular, cell-cell interactions in the Peyer's patches involving CD40 and/or CD80/CD86 have been implicated in germinal-center formation and IgA B-cell development. Also soluble factors, such as IL-4, IL-5, IL-6, and TGF$\beta$ may be critical for IgA B-cell differentiation *in vivo*. Here we report on some paradoxical findings with regard to IgA B-cell differentiation and specific mucosal immune responses that we have recently made using gene knockout mice. More specifically, we have investigated to what extent absence of CD4$^+$ T cells, relevant cytokines, or T-cell-B-cell interactions would influence IgA B-cell differentiation *in vivo*. Using CD4− or IL-4-gene knockout mice or mice made transgenic for CTLA4Ig, we found that, although specific responses were impaired, total IgA production and IgA B-cell differentiation appeared to proceed normally. However, a poor correlation was found between, on the one hand, GC formation and IgA differentiation and, on the other hand, the ability to respond to T-cell-dependent soluble protein antigens in these mice. Thus, despite the various deficiencies in CD4$^+$ T-cell functions seemingly intact IgA B-cell development was observed.

### INTRODUCTION

Mucosal surfaces are constantly exposed to a myriad of foreign antigens. To protect against pathogenic microorganisms or hostile compounds, such as toxins, the mucosa has developed effective barrier functions. For the maintenance of these barrier functions, secretory IgA (sIgA) is thought to play a particularly important role (McGhee et al., 1992). In the intestinal immune system, IgA B cells are derived from precursor cells in the Peyer's patches (PP) (Cebra and Shroff, 1994). After antigen stimulation, IgA lymphoblasts migrate from the PP via the mesenteric lymph node (MLN) and the thoracic lymph duct to the blood and eventually home back to the lamina propria of the gut mucosa. The development of normal PP morphology appears to be dependent on the local exposure to antigens in the intestine. Investigations of gnotobiotic or neonatal mice have demonstrated only a few IgA B cells in poorly developed PP (Cebra et al., 1980). After bacterial colonization of these mice, IgA B cells appear in great numbers in the PP, which now have

*Corresponding author.

increased in size and numbers. The IgA B cells in the PP largely represent dividing cells and the PP make up the largest pool of IgA-committed B cells in the body. Most of these $IgA^+$ B cells colocalize to germinal centers (GC) in the B-cell follicles of the PP. It is important to note that even without specific immunization, conventionally reared animals, in contrast to spleen and lymph nodes, exhibit large numbers of GC in PP (Butcher et al., 1982). One explanation for this may be that the IgA-committed B cells from PP commonly have specificities against phosphocholine, $\beta$2-1 fructosyl groups, or $\beta$ galactosyl groups, which are antigens that may be associated with the normal bacterial flora of the gut.

### GERMINAL CENTERS

Germinal centers are histologically defined areas containing rapidly dividing B lymphoblasts enmeshed in a network of follicular dendritic cells (FDC) and relatively few $CD4^+$ T cells (Liu and Banchereau, 1996). The GC is formed after antigen stimulation and reaches a maximum size after 10 to 14 days. A general view is that the GC reaction is dependent on the presence of $CD4^+$ T cells. Also in the gut mucosal immune system, $CD4^+$ T cells in PP are thought to play a critical role for the generation of IgA immune responses (Cebra et al., 1980). Studies in nude mice, lacking $\alpha\beta TCR^+$ cells but fully competent in B cells, have demonstrated impaired IgA B-cell differentiation (Cebra et al., 1980; Gottstein et al., 1993). In the PP, the GC contains 75-80% $IgA^+$ B cells, whereas in peripheral lymph nodes, very few $IgA^+$ B cells are seen (Butcher et al., 1982). Therefore, it is thought that the GC in PP differ intrinsically from those of spleen and lymph nodes in their content of particular kinds of antigen-presenting cells (APC), stromal cells, or regulatory T cells, and, hence, in the repertoire of cytokines and cell-cell interactions available for promoting isotype switching from IgM to IgA. Earlier experiments by Cebra et al. with local reovirus infection in germ-free mice demonstrated that the microenvironment of the PP may be selecting for direct isotype switching from IgM to IgA B-cell differentiation (Weinstein and Cebra, 1991). The prevailing theory on IgA B-cell differentiation argues for this process being a highly directed event strongly dependent on $CD4^+$ T cells (Strober and Ehrhandt, 1994). Recently, it was reported that a majority of the GC T cells may be phenotypically distinct $Thy1^-$ $CD4^+$ cells (Harriman et al., 1990; Zheng et al., 1996). Also accessory cells can provide necessary signals to drive IgA B-cell differentiation in PP (Schrader et al., 1990). Important accessory cells in the PP are dendritic cells (DC), both the interdigitating DC and the FDC in the GC. Whether there exists distinct functional subsets of DCs in the PP is currently unclear (Kelsall and Strober, 1996; Ruedl et al., 1996). Recent preliminary reports have suggested that dendritic cells isolated from PP or treated with IL-10 may favor Th2 differentiation and cytokine secretion of $CD4^+$ T cells.

### COSTIMULATION AND GC FORMATION

Earlier studies have clearly demonstrated that cell-cell interactions via the CD40L and CD28 on the activated T cell critically influence the formation of GC (Foy et al., 1994; Bluestone, 1995; Sharpe 1995). Whereas both CD40L and CD28 interactions appear to affect GC formation, isotype switching, and development of memory, there may also be differential effects on these events as well as on other events such as Ig hypermutation (Han et al., 1995). Moreover, it is also probable that these T-cell-B-cell interactions affect the development of the $CD4^+$ T-helper functions *in vivo* (Essen et al., 1995). We and others have found that oral immunization preferentially generates $CD4^+$ T cells of the Th2 type and experiments in $IL\text{-}4^{-/-}$ mice have shown that this cytokine may be required for intestinal immune responses against soluble protein antigens (Xu-Amano et al., 1993; Vajdy et al., 1995). Recent studies have indicated that expression of CD40L on activated T cells may be more strongly associated with Th2 rather than Th1 development in $CD4^+$ T cells (Freeman et al., 1995). Therefore, T cells that are high in CD40L expression may be one of the defining features of the IgA-promoting environment in PP.

### REQUIREMENT FOR CD4$^{+}$ T CELLS IN IgA B-CELL DIFFERENTIATION

For IgA B-cell differentiation, CD4$^{+}$ T cells are thought to play a critical role as helper cells producing soluble factors, such as TGF$\beta$, IL-5, and IL-6, as well as engaging in contact-dependent cell-cell interactions (Strober and Ehrhardt, 1994). Whereas TGF$\beta$ acts as a possible switch factor early in IgA B-cell differentiation, IL-5 and IL-6 act later, on already committed IgA$^{+}$ B cells. Since most of the cytokines that have been implicated in IgA B-cell development are truely pleiotropic and produced by macrophages, fibroblasts, epithelial cells as well as B and T lymphocytes in the mucosa, our understanding of the interactions between these cells and the IgA B-cell progeny is still poor. The introduction of transgene- and gene-targeting techniques has provided powerful new tools with which to address complex issues such as the regulation of mucosal immune responses and IgA B-cell development (Lycke et al., 1995). In our laboratory, we have taken advantage of these mice to address some of the critical questions in mucosal immunolgy.

### PARADOXES IN IgA B-CELL DIFFERENTIATION IN GENE KNOCKOUT MICE

In this review, we will discuss some of the paradoxes of IgA B-cell immunity that we have observed in mice made genetically deficient for critical T-cell functions. Our experimental model system uses soluble protein antigens such as keyhole limpet hemocyanin (KLH) or ovalbumin (OVA) given orally together with cholera toxin (CT) adjuvant for induction of intestinal immune responses. We have studied IgA B-cell differentiation and the ability to respond to oral immunizations in CD4 gene-targeted (CD4$^{-/-}$) mice, IL-4$^{-/-}$, and IL-6$^{-/-}$ mice or mice overexpressing the CTLA4-H$\gamma$1 protein, which blocks T-cell-B-cell interaction via the CD80 and CD86 surface molecules. The latter CTLA4-H$\gamma$1 transgenic mice (TG) have previously been reported to lack GC formation and isotype switch differentiation in systemic tissues following specific immunizations with T-cell-dependent antigens (Lane et al., 1994).

Using normal and CD4$^{-/-}$ mice, we asked whether mucosal immune responses and IgA B-cell differentiation required the presence of CD4$^{+}$ T-helper cells (Hörnquist et al., 1995). Quite unexpectedly we found that CD4$^{-/-}$ mice had numerous GC in PP and other gut-associated lymphoid tissues (GALT), whereas only few GCs were evident in spleen and lymph nodes. Membrane IgA$^{+}$ B cells were found to colocalize to GC areas and CD4$^{-}$CD8$^{-}$ double-negative (DN) CD3$^{+}$ T cells had replaced CD4$^{+}$ T cells in the follicular areas of the PP. A recent study analyzing TCR$\alpha^{-/-}$ mice also reported on GC formation in PP despite the lack of classical $\alpha\beta$TCR$^{+}$ cells, suggesting that the lymphoid tissue in PP may be differently regulated from that in peripheral lymph nodes (Dianda et al., 1996). Alternatively, there may exist microbial antigens in the gut microenvironment that can drive GC formation with a limited requirement for $\alpha\beta$TCR$^{+}$ CD4$^{+}$ T-cell help. The latter notion is supported by the finding that germ-free TCR$\alpha^{-/-}$ mice had less GC in PP than did conventionally reared mice. However, at variance with our findings, in CD4$^{-/-}$ mice, the latter study suggested that expression of CD4 was important for GC formation in PP (Hörnquist et al., 1995; Dianda et al., 1996). Nevertheless, at present, we must conclude that the relationship between CD4$^{+}$ T cells and the formation of GC in PP is poorly understood.

The CD4$^{-/-}$ mice had normal levels of IgA-producing cells in GALT and gut lavage contained unaltered levels of total IgA (Hörnquist et al., 1995). In spite of what appeared to be adequate T-cell help for IgA B-cell differentiation, CD4$^{-/-}$ mice did not respond with Ag-specific intestinal or serum IgA following oral immunization with CT. Moreover, perorally immunized CD4$^{-/-}$ mice were completely unprotected against CT-induced diarrhea, whereas normal mice were well-protected and demonstrated high levels of antitoxin IgA in gut lavage. Thus, paradoxically, whereas IgA B-cell differentiation appeared to proceed normally in CD4$^{-/-}$ mice, specific gut mucosal immune responses were impaired in the absence of CD4+ T cells. A poor correlation between GC formation and presence of serum antibody has previously been reported by Stedra and Cerny (1994), and based on our findings, it seems reasonable to assume that the presence of GC in PP does not constitute proof of the ability to respond to oral immunization with T-cell-dependent antigens.

TABLE I Levels of Total IgA in Gut Lavage of CTLA4-Hγ1 TG Mice[a]

| Experiment | Total IgA | |
|---|---|---|
| | C57Bl/6 | CTLA4-Ig |
| 1 | 2.8 ± 0.3 | 2.8 ± 0.1 |
| 2 | 3.1 ± 0.3 | 3.0 ± 0.2 |

[a]Gut lavage was performed and the content of total IgA antibodies determined by ELISA. The results are given as mean $\log_{10}$ titers ± SD of two experiments with five to seven animals.

### IgA B-CELL DIFFERENTIATION IN THE IL-4 KNOCKOUT MOUSE

IL-4 has been found responsible for the generation of Th2 functions in CD4+ T cells (Reiner and Seder, 1995). After oral immunizations in normal mice, Th2 cells were selectively induced, suggesting that IL-4 may be of particular interest for the control of mucosal immune responses (Xu-Amano et al., 1993; Vajdy et al., 1995). Moreover, IL-4 has been ascribed a role in IgA B-cell differentiation, possibly as a switch factor, in recent studies (Wakatsuki and Strober, 1993; Strober and Ehrhardt, 1994). However, we observed that, paradoxically, total IgA levels in gut lavage and serum as well as total numbers of IgA-containing cells in lamina propria were unaltered in IL-4$^{-/-}$ mice as compared to wild-type mice (Vajdy et al., 1995). Thus, it appeared that IgA B-cell differentiation was unaffected by the total absence of IL-4 or Th2 cells. Furthermore, a striking and consistent finding was that PP in the IL-4$^{-/-}$ mice demonstrated no or poor GC reactions, not even after oral immunization. By contrast, and similar to wild-type mice, GCs were prominent in mesenteric lymph nodes and spleen. Also, systemic immune responses were unaltered in IL-4$^{-/-}$ mice as compared to wild-type mice. Therefore, we believe, that whereas IL-4 is not critical for stimulation of immune responses at systemic sites, the gut intestinal tract and probably also mucosal immunity in general is strongly dependent on IL-4 or Th2 cells. In support of this notion, it was found in GC in human tonsils that IL-4 was the only cytokine constitutively expressed (Butch et al., 1993). An unanswered question is why IL-4 appears to be more critical for GC formation in PP as compared to peripheral lymph nodes.

### MUCOSAL IMMUNITY IN IL-6 KNOCKOUT MICE

In IL-6$^{-/-}$ mice we observed normal numbers and appearances of GC in PP to which IgA+ B cells colocalized. This result was indicative of that IgA-isotype differentiation may be occurring *in situ* in PP and lamina propria even in the absence of IL-6 (Bromander et al., 1996). Also, the ability to respond with mucosal IgA following oral and intranasal immunization with specific antigen, KLH or OVA, in the presence of CT adjuvant or to live *Helicobacter felis* infection was similar in IL-6$^{-/-}$ and wild-type mice. CT exerted strong and comparable adjuvant functions in IL-6$^{-/-}$ and wild-type mice. We concluded that, although IL-6 has been ascribed a crucial role for terminal differentiation of IgA B cells *in vitro*, we found no evidence to support the notion that IL-6 is critically required for IgA B-cell development or specific mucosal IgA responses *in vivo*.

### INVOLVEMENT OF CD40L INTERACTIONS

Recent studies *in vitro* using LPS- or CD40L-induced B-cell stimulation have provided evidence suggesting that IgA B-cell differentiation may depend on cell-cell interactions (Rousset et al., 1991; Ehrardt et al., 1996). The data suggest that whereas IgA-switch

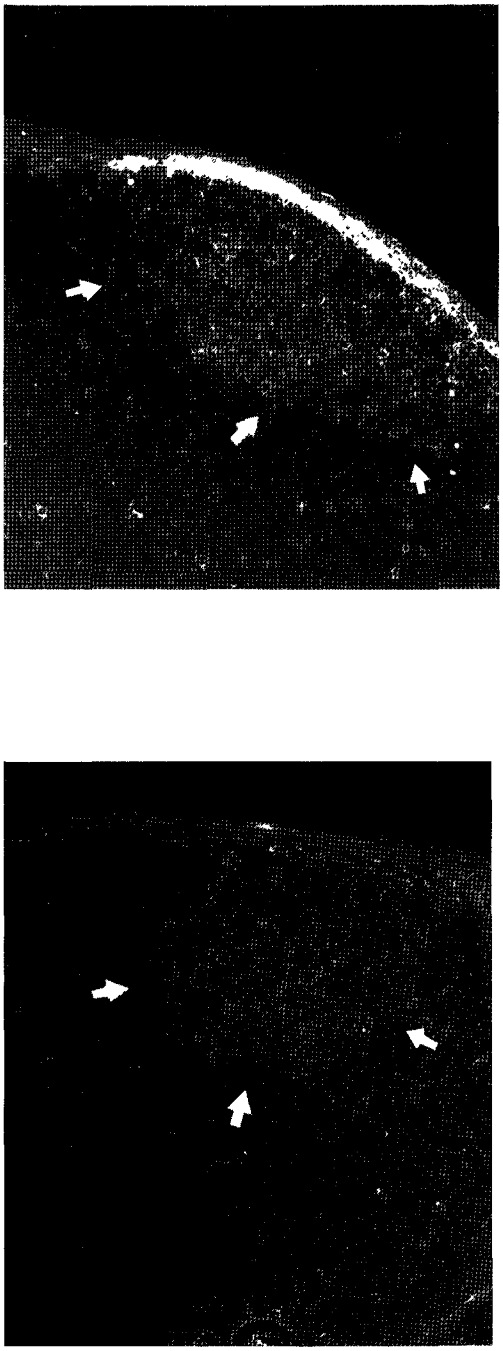

FIGURE 1 Micrographs demonstrating presence of germinal centers in the PP of CTLA4-H$\gamma$1 mice. Immunofluorescense labeling of PNA-binding cells constituting a GC (arrows) in (A) wild-type mice and (B) in CTLA4-H$\gamma$1 transgenic mice.

differentiation may proceed in the presence of soluble T-cell factors, that is, cytokines, terminal differentiation of $IgA^+$ B cells may critically depend on B-cell interaction with activated CD40L-expressing T cells. $IgA^+$ B cells, in contrast to $IgA^-/IgM^+$ B cells, were strikingly more responsive to T-cell dependent activation, that is, CD40L interaction, than to T-cell-independent activation via membrane cross-linking of receptors. Thus, the close interaction with the activated T cell most probably is a necessary precondition for terminal IgA B-cell differentiation *in vivo*. However, there was no difference in responsiveness between PNA-high B cells isolated from GC of PP and PNA-low-lamina-propria $IgA^+$ B cells, suggesting that CD40L expression may not be sufficient to explain the regulatory role of $CD4^+$ T cells in these two locations. Nevertheless, collectively, the data provide evidence that costimulation is playing a pivotal role in IgA B-cell differentiation.

### IgA B-CELL DIFFERENTIATION IN CTLA4-Ig-TRANSGENIC MICE

Because it can be postulated that IgA differentiation is highly dependent on activated T cells, we investigated to what extent cell-cell interactions between the B cell and the T cell would influence IgA B-cell differentiation *in vivo*. To this end, we used the recently developed CTLA4-H$\gamma$1 TG mice, which were reported to lack GC and exhibit poor B-cell isotype switching following systemic immunizations with soluble protein antigens (Lane et al., 1994). The transgenic mice overexpress the CTLA-4Ig-protein, which binds both CD80 and CD86 and blocks the interaction with the CD28/CTLA-4 receptors. We found that in unimmunized CTLA4-H$\gamma$1 TG mice, total serum IgA levels were normal, whereas total IgG levels were significantly reduced. Also, total IgA in gut lavage was normal (Table I). Whereas Lane et al. (1994) reported that CTLA4-H$\gamma$1 TG mice lacked GC in spleen and lymph nodes, immunohistochemical analysis of frozen tissue sections of gut mucosa clearly demonstrated GC formation in PP of these

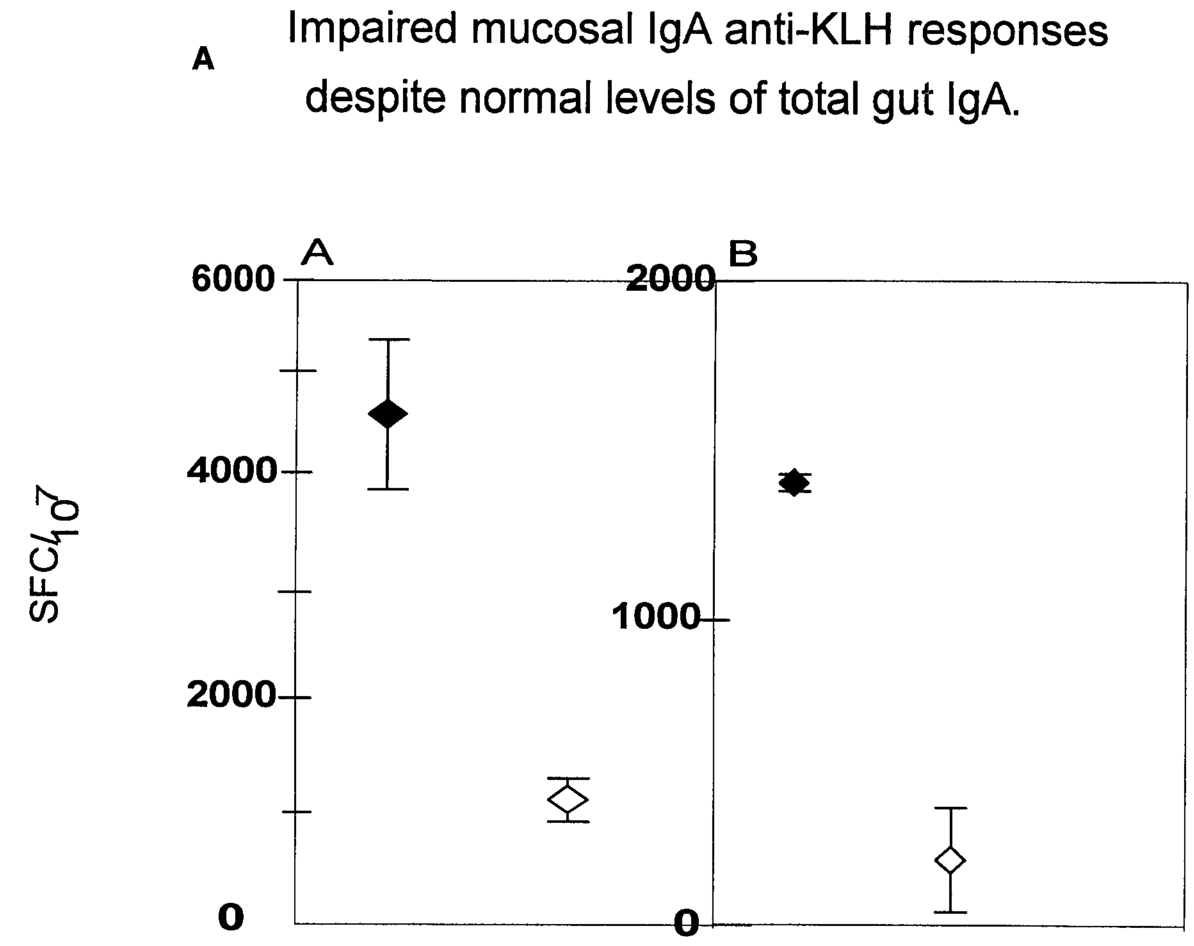

FIGURE 2 Impaired-specific mucosal immune response in CTLA4-Hγl transgenic mice after oral immunization with KLH and cholera toxin. (A) KLH-specific IgA spot forming cells/$10^7$ isolated lymphoid cells in lamina propria in C57Bl/6 (solid diamonds) and in CTLA4-Hγl (open diamonds) mice as determined by ELISPOT. (B) Total KLH-specific spot forming cells/$10^7$ isolated lymphoid cells in the spleen. The results are given as mean value ± SEM of two experiments. The differences in responsivenes between transgenic and the wild-type mice are statistically significant ($p < 0.01$).

TABLE II Unaltered T-Cell Priming Efficiency of Oral Immunizations in CTLA4-Hγl TG Mice[a]

| Mice | KLH | | Anti-CD3 | | Anti-CD3 + anti CD28 | |
|---|---|---|---|---|---|---|
| | c.p.m. | SI | c.p.m. | SI | c.p.m. | SI |
| C57Bl/6 | 2276 ± 1366 | 2.4 ± 1.1 | 2891 ± 649 | 5.4 ± 1.8 | 32,802 ± 9,757 | 55 ± 13 |
| CTLA4-Ig | 2859 ± 1294 | 3.0 ± 1.5 | 6674 ± 4936 | 24.6 ± 4.3 | 47,199 ± 11,452 | 97 ± 19 |

[a]After three peroral immunizations with KLH + CT adjuvant single-cell suspensions from the spleen were prepared and cultured for 3 days in the presence or abscence of KLH, anti-CD3, or anti-CD3 + anti-CD28 mAb. Cell proliferation was determined on day 3 by [$^3$H TdR] uptake and the results were expressed as mean c.p.m. values ± SD of triplicate cultures. Stimulation indices (SI) were calculated and are given as means ± SD of triplicate cultures. This is one representative experiment of four.

mice (Figure 1). In accordance with previous findings in normal mice, the IgA$^+$ B cells colocalized to the GC areas in PP. Thus, similar to the IL-4$^{-/-}$ mice, we found unaltered total IgA concentrations in serum and intestinal lavage, but, in contrast to these mice, we observed many GC in PP of CTLA4-H$\gamma$l TG mice.

Previously, it was reported that systemic antibody responses to T-cell-dependent antigens were significantly impaired in the CTLA4-H$\gamma$l TG mice (Lane et al., 1994). As the lack of an IgG response appeared to be directly related to the absence of systemic GC formation, we tested whether mucosal IgA responses required less T-cell-B-cell cooperation to generate mucosal IgA immunity. Following oral immunization with KLH plus CT-adjuvant local IgA as well as systemic anti-KLH responses in CTLA4-H$\gamma$l, TG mice were strongly impaired (Figure 2). Thus, paradoxically, we observed unimpeded total IgA levels in the gut lamina propria, and GC formation and IgA B-cell isotype-switch differentiation in PP, and yet the response to both KLH and CT were severely impaired in the CTLA4-H$\gamma$l TG mice.

Moreover, in contrast to IL-4$^{-/-}$ mice (Vajdy et al., 1995), we found that oral immunizations efficiently primed T cells in the CTLA4-H$\gamma$l TG mice. As illustrated in Table II, we obtained comparable responses to recall antigen in spleen T-cell cultures from CTLA4-H$\gamma$l TG and normal C57Bl/6 mice following oral immunizations with KLH plus CT adjuvant. Also, the ability of splenic T cells from CTLA4-H$\gamma$l TG mice to respond to anti-CD3 and anti-CD28 costimulation was unaltered as compared to normal mice (Table 2). This finding agrees well with previous reports demonstrating unaltered ability to induce T-cell responses in the CTLA4-H$\gamma$l TG mice (Ronchese et al., 1994), whereas T-cell help for IgA B-cell differentiation after specific oral immunizations appears to be inhibited.

### CONCLUDING REMARKS

In conclusion, the CTLA4-H$\gamma$l TG mice, as well as CD4$^{-/-}$ and IL-4$^{-/-}$ mice, have shown impaired mucosal immune responses to oral immunizations with the highly immunogenic combination of KLH and CT adjuvant. Although, specific responses were impaired, total IgA production and IgA B-cell differentiation appeared to proceed normally in these mice, despite the various deficiencies in CD4+ T-cell functions. Thus, paradoxically, we found poor correlation between, on the one hand, GC formation and IgA differentiation and, on the other hand, the ability to respond to T-cell-dependent soluble protein antigens. Experiments are ongoing to resolve these conflicting findings.

### *References*

Bluestone J.A. (1995). New perspectives of CD28-B7-mediated T-cell costimulation. Immunity. **2**: 555-559.

Bromander A., Ekman L., Kopf M., Nedrud J., and Lycke N. (1996). IL-6-deficient mice exhibit normal mucosal IgA responses to local immunizations and *Helicobacter felis* infection. J. Immunol. **156**: 4290-4297.

Butch A.W., Chung G.H., Hoffmann J.W., and Nahm M.H. (1993). Cytokine expression by germinal center cells. J. Immunol. **150**: 39-47.

Butcher E.C., Rouse R.L., Nottenberg C.N., Hardy R.R., and Weissman I. (1982). Surface phenotype of Peyer's patch germinal centers: Implications for the role of germinal centers in B-cell differentiation. J. Immunol. **129**: 2698.

Cebra J.J., Gearhart P.J., Halsey J.F., Hurwitz J.L., and Shahin R.D. (1980). Role of environmental antigens in the ontogeny of the secretory immune response. J. Reticuloendothel. Soc. **28**: 61-71.

Cebra J.J., and Shroff K. (1994). Peyer's patches as inductive sites for IgA comittment. In Handbook of Mucosal Immunology, Ogra P., Strober W., Mestecky J., McGhee J., Lamm M., and Bienenstock J., Eds. (San Diego: Academic Press), pp. 151-158.

Dianda L., Gulbranson-Judge A., Pao W., Hayday A., MacLennan I., and Owen M. (1996). Germinal center formation in mice lacking ab T cells. Eur. J. Immunol. **26**: 1603-1607.

Ehrardt R., Gray B., Duchmann R., Inman J., and Strober W. (1996). Reciprocal regulation of mucosal surface IgA+ B cells by Ig receptor cross-linking and CD40 ligand. J. Immunol. **157**: 1397-1405.

Essen D., Kikutani H., and Gray D. (1995). CD40 ligand-transduced co-stimulation of T cells in the development of helper function. Nature **378**: 620-623.

Foy T.M., Laman J.D., Ledbetter J.A., Aruffo A., Claassen E., and Noelle R. (1994). gp-39-CD40 interactions are essential for germinal center formation and the development of B cell memory. J. Exp. Med. **180**: 157-163.

Freeman G.J., Boussiotis V.A., Anumanthan A., Bernstein G.M., Ke X.Y., Rennert P.D., Gray G.S., Gribben J.G., and Nadler L.M. (1995). B7-1 and B7-2 do not deliver identical costimulatory signals, since B7-2 but not B7-1 preferentially costimulates the initial production of IL-4. Immunity **2**: 523-532.

Gottstein B., Deplazes P., and Tanner I. (1993). In vitro synthezised immunoglobulin A from nu/+ and reconstituted nu/nu mice against a dominant surface antigen of Giardia lamblia. Parasitol. Res. **79**: 644.

Han S., Hathcock K., Zheng B., Kepler T. Hodes R., and Kelsoe G. (1995). Cellular interaction in germinal centers. Roles of CD40 ligand and B7-2 in established germinal centers. J. immunol. **155**: 556-567.

Harriman G.R., Lycke N.Y., Elwood L.J., and Strober W. (1990). T lymphocytes that express CD4 and the ab-T cell receptor but lack Thy-1. J. Immunol. **145**: 2406-2414.

Hörnquist E., Ekman L., Grdic D., Schön K. and Lycke N. (1995). Paradoxical IgA immunity in CD4-deficient mice: Lack of cholera toxin specific protective immunity despite normal gut mucosal IgA differentiation. J. Immunol. **155**: 2877-2887.

Kelsall B.L., and Strober W. (1996). Distinct populations of dendritic cells are present in the subepithelial dome and T cell regions of the murine Peyer's patch. J Exp. Med. **183**: 237-247.

Lane P., Burdet C., Hubele S., Schedegger D., Muller U., McConell F., and Kosco-Vilbois M. (1994). B cell function in mice transgenic for mCTLA4-Hg1: Lack of germinal centers correlated with poor affinity maturation and class switching despite normal priming of CD4+ T cells. J. Exp. Med. **179**: 819-830.

Liu Y.J., and Banchereau J. (1996). The paths and molecular controls of peripheral B-cell development. Immunologist **4**: 55-66.

Lycke N., Bromander A.K., Ekman L., Grdic D., Hörnquist E., Kjerrulf M., Kopf M., Kosco-Vilbois M., Schön K., and Vajdy M. (1995). The use of knock-out mice in studies of induction and regulation of gut mucosal immunity. Mucosal Immunol. Update **3**, 000-000.

McGhee J.R., Mestecky J., Dertzbaugh M.T., Eldridge J.H., Hirasawa M., and Kiyono H. (1992). The mucosal immune system: From fundamental concepts to vaccine development. Vaccine **10**: 75.

Reiner S.L., and Seder R.A. (1995). T helper cell differentiation in immune response. Curr. Opin. Immunol. **7**: 360-366.

Ronchese F., Hausmann B., Hubele S., and Lane P. (1994). Mice transgenic for a soluble form of murine CTLA4 show enhanced expansion of antigen-specific CD4+ T-cells and defective antibody production in vivo. J. Exp. Med. **179**: 809-817.

Rousset F., Gracia E., and Banchereau J. (1991). Cytokine-induced proliferation and immunoglobulin production of human B lymphocytes triggered through their CD40 antigen. J. Exp. Med. **173**: 705-710.

Ruedl C., Rieser C., Böck G., Wick G. and Wolf H. (1996). Phenotypic and functional characterization of CD11c+ dendritic cell population in mouse Peyer's patches. Eur. J. Immunol. **26**: 1801-1806.

Schrader C.E., George A., Kerlin R.L., and Cebra J.J. (1990). Dendritic cells support production of IgA and other non-IgM isotypes in clonal microculture. Int. Immunol. **2**: 563-570.

Sharpe A. (1995). Analysis of costimulation in vivo using transgenic and knockout mice. C. O. Immunol. **7**: 389-395.

Stedra J., and Cerny J. (1994). Distinct pathways of B cell differentiation. J. Immunol. **152**: 1718.

Strober W., and Ehrhardt R. (1994). Regulation of IgA B cell development. In Handbook of Mucosal Immunology, Ogra P., Strober W., Mestecky J., McGhee J., Lamm M., and Bienenstock J., Eds. (San Diego: Academic Press), pp. 159-176.

Vajdy M., Kosco-Vilbois M., Kopf M., Köhler G., and Lycke N. (1995). Impaired mucosal immune responses in interleukin 4-targeted mice. J. Exp. Med. **181**: 41-53.

Wakatsuki Y., and Strober W. (1993). Effect of downregulation of germline transcripts on immunoglobulin A isotype differentiation. J. Exp. Med. **178**: 129-138.

Weinstein P.D., and Cebra J.J. (1991). The preference for switching to IgA expression by Peyer's patch germinal center B cells is likely due to the intrinsic influence of their microenvironment. J. Immunol. **147**: 4126-4135.

Xu-Amano J., Kiyono H., Jackson R., Stats H.F., Fujihashi K., Burrows P.D., Elson C.O., Pilla S., and McGhee J.R. (1993). Helper T cell subsets for immunoglobulin A responses: Oral immunization with tetanus toxoid and cholera toxin as adjuvant selectively induces Th2 cells in mucosa associated tissues. J. Exp. Med. **178**: 1309.

Zheng B., Han S., and Kelsoe G. (1996). T helper cells in murine germinal centers are antigen-specific emigrants that downregulate Thy-1. J. Exp. Med. **184**: 1083-1091.

